# Commentary: Is obesity associated with taste alterations? a systematic review

**DOI:** 10.3389/fendo.2023.1282276

**Published:** 2024-01-19

**Authors:** Marco Alessandrini, Alessandra Vezzoli, Simona Mrakic-Sposta, Sandro Malacrida, Alessandro Micarelli

**Affiliations:** ^1^ Department of Clinical Sciences and Translational Medicine, Ear-Nose-Throat (ENT) Unit, University of Rome Tor Vergata, Rome, Italy; ^2^ Institute of Clinical Physiology, National Research Council (CNR), Milan, Italy; ^3^ Institute of Mountain Emergency Medicine, Eurac Research, Bolzano, Italy; ^4^ Unit of Neuroscience, Rehabilitation and Sensory Organs, UNITER ONLUS, Rome, Italy

**Keywords:** obesity, leptin, smell, taste, ghrelin

## Introduction

We read with interest the systematic review “Is obesity associated with taste alterations? a systematic review” by Peinado and colleagues investigating the main outcomes of nineteen papers published in the field of obesity and taste perception (1). The topic is of interest considering the burdening of the obesity outbreak (2) and the works pointed to reduce the epidemic have almost failed (3). Emerging results highlighted that the taste system – together with the smell – could be engaged in diverse metabolic processes (for example appetite control and energy homeostasis) possibly influencing body weight and health (4, 5). Some of the feedback-forward processes have been postulated to also rely on different modulators such as leptin, ghrelin, insulin and serum glucose which have been demonstrated to be entangled in the activity of sweet-sensing taste cells (6–8) and to modulate chemosensory pathways at central levels (8, 9).

## A research update in taste-obesity association

In this vision, our research group published in February 2023 an observational study in which taste function – together with serum levels of the above mentioned biochemical regulators, food frequency questionnaires scores and anthropometric parameters – have been investigated in 60 leans (LS), 39 overweight (OW), 18 stage I obesity, and 20 stage II obesity participants (10). These participants were tested for the four basic taste components (sweet, sour, salty, and bitter) in ascending concentrations by means of the taste test - consisting of filter paper strips (“Taste Strips”, Burghart, Wedel, Germany) (11) - and by using a randomized procedure on both sides of the anterior third of the tongue, resulting in a total of 32 trials (12).

A significant decrease in total and all subtests taste scores in stage II obesity participants was found when compared with LS. Likewise, a significant reduction in, salty, bitter and total taste scores was showed in stage II when compared with OW subjects. Further, a stepwise significant increase in leptin, insulin, serum glucose and a decrease in ghrelin was found in stage I and II obesity participants when compared with OW and LS. Interestingly, an inverse association was found between total taste score and leptin, levels of monounsaturated fatty acids taken with diet and level of visceral fat.

## Discussion

As remarked by Peinado and colleagues in their seminal review (1), besides the relationship between gustatory sensitivity and body mass index (BMI) has been extensively examined with non univocal findings in the last decades, it has been continuously evidenced - in line with the results of the work by Micarelli et al. - that a certain degree of perception reduction of different taste components (increase in taste threshold) and weakened sense of taste could be gathered with the BMI increase (13–16). These aspects have been also confirmed for the (less grasped understood) sense of smell (17, 18) and indirectly corroborated by those studies depicting that bariatric surgery and/or specific nutritional interventions may revert such processes (19–21).

At hormonal levels, a stepwise increase in insulin and leptin and a reduction in ghrelin, with possible consequences on the imbalance of the gustatory signaling and food reward (22), has been found in subjects affected by OW and stages I–II obesity. For instance, beyond leptin has been demonstrated to enhance appetite in response to its reduced or absent circulating levels (23), this regulator could also exert its activity via binding to its obese receptor in type II taste buds cells and interfere with local K_ATP_ channels (24). This could result in a dampened sweet response signal transduction to the afferent nerve fiber in the taste cell and in a reduced perception of sweet quality (7). Considering that serum leptin concentration is markedly associated with BMI (8) and that it has been found in our study to be inversely associated with total taste scores, it could be pointed as one of the underpinnings of the sweet sensitivity decrease often found in patients with obesity (25). However, obesity is a complex process and findings of the review of Peinado and colleagues may also be accounted by further phenomena. First of all, visceral fat – rather than subcutaneous one - is extremely metabolically active (26) and may impact on the progression of metabolic disorders by fostering the pro-inflammatory milieu (27), possibly affecting the decrease of taste buds. This is also supported by previous results in mice which found that chronic low-grade inflammation sustained by obesity could decrease the number of taste buds in gustatory tissues (6). Secondly, obese individuals follow a dietary regimen enriched in sugar and fat which have been associated – as in our study - with a dampened taste stimuli sensitivity, thus impacting of dietary choices and promoting food intake (6, 25, 28, 29). Previous studies - indeed - observed significant fat perception gain after a low-fat dietary regimen (30, 31). They indicated that taste sensitivity changes to fatty acids might be the consequence of taste adjustment to a high-fat dietary regimen and may foster the fat intake surplus due to a weakened gustatory response to fatty acids among those subjects who routinely follow a diet highly enriched in fat, as it happens in overweight/obese individuals (31). These phenomena have been ascribed to a downregulation in the expression of specific subunits of sensing G-protein coupled receptors of diverse nourishing substances, which, in light of their cross-sensitivity, may be responsible of the downward phenomena regarding also other taste components sensitivity (32).

Although the systematic review properly asserted that many biases are influencing the results explanation of studies in this field, we believe that results of our work could further enlarge the discussion of Peinado and colleagues. Indeed, some efforts in elucidating the association between taste weakness and obesity by means of standardized testing methods and biochemical assays has been achieved and hypothesized a certain framework of interactions which may partially underlie taste (and smell) dampening processes along the obesity development.

## Author contributions

MA: Conceptualization, Funding acquisition, Resources, Writing – original draft, Writing – review & editing. AV: Data curation, Methodology, Writing – review & editing. SM: Methodology, Supervision, Writing – review & editing. SM: Data curation, Supervision, Writing – review & editing. AM: Data curation, Methodology, Writing – original draft, Writing – review & editing.
